# Bayesian Hierarchical Spatiotemporal Prediction of Multi-pathogen Outbreaks: Integrating Mobility and Environmental Exposures

**DOI:** 10.7759/cureus.107576

**Published:** 2026-04-23

**Authors:** Hakeem Adekunle, Oladimeji S Adewuyi, Kayode Okunola, Olalekan T Balogun

**Affiliations:** 1 Statistics, Georgia State University, Atlanta, USA; 2 Microbiology, University of Ibadan, Ibadan, NGA

**Keywords:** disease surveillance, emerging pathogen, environmental exposure, human mobility, mobility, spatiotemporal

## Abstract

Introduction: Infectious disease outbreaks remain a persistent global health burden, particularly as populations experience the concurrent circulation of multiple infectious agents that interact across space and time. Forecasting such complex epidemic systems requires models that can capture shared transmission mechanisms, pathogen-specific dynamics, and uncertainty arising from incomplete surveillance.

Methods: In this study, we develop a unified Bayesian hierarchical spatiotemporal framework for predicting multi-pathogen outbreak trajectories while integrating human mobility patterns, environmental exposures, and structured reporting uncertainties. We conducted a comprehensive simulation experiment to evaluate the model’s ability to recover known parameters, distinguish pathogen-specific transmission effects, and generate calibrated forecasts under varying levels of reporting noise and spatial heterogeneity. We further applied the method to CDC FluView surveillance weekly data from the United States, spanning January 2017 to December 2025.

Results: In the simulation study, the model showed good parameter recovery under different levels of reporting noise and spatial heterogeneity, with stable estimates and satisfactory convergence. The model effectively distinguished pathogen dynamics, with posterior means for baseline incidence ($\alpha_{1}$ and $\alpha_{2}$ at -0.74 and -0.87). Human mobility ($\eta$ = 0.35, 95% CI: 0.06-0.72) was a significant driver of spatial transmission, while overdispersion parameters ($\phi_{1}$ = 11.61, $\phi_{2}$ = 5.74) accounted for variability beyond the mean structure. Diagnostic Rhat ​values near 1 confirmed model convergence and robust chain mixing. In the real data application, influenza showed a higher baseline incidence than measles ($\alpha_{1}$ = -0.74, $\alpha_{2}$ = -0.87), temperature had a positive effect on transmission ($\beta$ = 0.19), while humidity effects were weaker and more uncertain, and the mobility parameter ($\eta$ = 0.35) indicated that human movement contributed to spatial spread; influenza also exhibited greater variability ($\phi_{1}$ = 11.61 vs $\phi_{2}$ = 5.74), and the model captured seasonal patterns while closely tracking the observed incidence over time.

Conclusions: Across all scenarios, the model demonstrated robust parameter recovery, reduced bias in reproduction number estimates, and improved predictive accuracy relative to conventional compartmental and independent-pathogen Bayesian models. This performance was consistent in both simulation and real data settings, where the model distinguished pathogen-specific dynamics, captured the contribution of human mobility to spatial transmission, and accounted for variability in case counts through overdispersion. The results support the use of this approach for stable inference and reliable forecasting in complex multi-pathogen systems.

## Introduction

Infectious disease outbreaks remain a persistent global health burden, particularly as populations experience concurrent circulation of multiple infectious agents such as influenza viruses, respiratory syncytial virus (RSV), and severe acute respiratory syndrome coronavirus 2 (SARS-CoV-2). SARS-CoV-2, dengue, and other arboviruses pose substantial challenges to public-health preparedness and outbreak response [1]. Multi-pathogen epidemics are characterized by inter-pathogen interactions (competition, facilitation, or interference), shared environmental drivers, and overlapping host networks; these features complicate inference and forecasting when models treat pathogens independently [1]. Accurate prediction of joint outbreak trajectories is essential for anticipating healthcare demand, optimizing surveillance priorities, and guiding timely interventions. Two streams of data have become increasingly available and relevant for epidemic modeling. First, high-resolution human mobility data derived from mobile devices, transportation systems, and aggregated telecom records capture population movement patterns that strongly shape the spatial dissemination of infections [2]. Numerous studies during the COVID-19 pandemic showed that mobility metrics both explain early seeding events and help evaluate the effect of non-pharmaceutical interventions, although their predictive value can vary with context, representativeness, and temporal scale [3].

Second, environmental exposures including temperature, humidity, precipitation, ultraviolet radiation, and air pollution indices modulate pathogen survival, vector dynamics, and host susceptibility and thus act as important seasonal and spatial drivers of transmission [4,5]. Despite recognition of these drivers, operational forecasting systems frequently incorporate mobility or environment as ad hoc covariates rather than as integrated dynamic components. A persistent obstacle to robust outbreak prediction is reporting uncertainty within routine surveillance. Case counts are routinely biased by under-reporting, reporting delays, heterogeneity in testing practices, and misclassification; failing to account for these will systematically distort estimates of incidence and forecast uncertainty [6]. Nowcasting and correction methods can mitigate these problems, but existing implementations are often pathogen-specific and do not scale readily to joint multi-pathogen frameworks [6].

Bayesian hierarchical spatiotemporal models provide a coherent probabilistic framework to integrate heterogeneous data sources, represent latent epidemiological processes, and propagate multiple levels of uncertainty [7]. Hierarchical priors enable borrowing of strength across pathogens, spatial units, and time periods, while spatial and temporal random effects capture residual dependencies. Recent methodological advances (e.g., integrating latent process formulations, Integrated Nested Laplace Approximation (INLA), and scalable Markov Chain Monte Carlo (MCMC) approaches) make it feasible to fit high-dimensional joint models for surveillance data [7]. Nonetheless, published applications have largely focused on single pathogens or on joint spatial analysis without dynamic inclusion of mobility networks as transmission drivers, explicit environmental forcing at appropriate spatiotemporal resolutions, or integrated observation models for reporting error. This paper addresses that gap by developing and evaluating a Bayesian hierarchical spatiotemporal framework for jointly predicting outbreak trajectories of multiple pathogens. The model explicitly links latent transmission processes across pathogens through shared and pathogen-specific spatiotemporal components, integrates dynamic mobility networks as spatial coupling terms, incorporates high-resolution environmental covariates as time-varying transmission modifiers, and embeds an observation submodel to correct for underreporting and reporting delays. We aim to formulate a flexible, interpretable probabilistic model that captures pathogen-level and shared drivers of transmission; demonstrate how mobility and environmental exposures can be incorporated mechanistically into the spatiotemporal transmission kernel; develop practical strategies for modeling surveillance reporting processes within the joint framework; and evaluate predictive performance and uncertainty quantification via simulation and retrospective case studies. By unifying pathogen interactions, mobility connectivity, environmental forcing, and surveillance uncertainty in a single Bayesian framework, the proposed approach seeks to improve real-time situational awareness and to provide decision-relevant probabilistic forecasts for settings where multiple pathogens co-circulate and surveillance is noisy and incomplete.

## Materials and methods

This study was conducted using publicly available secondary datasets obtained from the CDC, including influenza data from FluView and measles incidence data from CDC national surveillance systems. In this study, we develop a Bayesian hierarchical spatiotemporal model to predict outbreak trajectories of multiple co-circulating pathogens jointly. The proposed framework is designed to explicitly incorporate human mobility patterns, environmental exposures, and uncertainties in case reporting. Our main goal is to understand how each disease spreads on its own, while also leveraging shared patterns across space and time and potential interactions between diseases to improve prediction and better quantify uncertainty. All analyses were conducted in R software version 4.2 (R Foundation for Statistical Computing, Vienna, Austria, https://www.r-project.org/) using the "rstan" package version for Bayesian inference [8].

Model structure

Let $Y^{p}_{i,t}$ denote the number of reported cases for pathogen $p$ in spatial unit $i$ at time $t$, where $p = 1, \ldots, P$, $i = 1, \ldots, N$, and $t = 1, \ldots, T$. We model these counts using a negative binomial distribution to account for overdispersion commonly observed in epidemiological data.

$Y_{i,t}^{(p)} \sim \mathrm{NegBin}\left(\lambda_{i,t}^{(p)} \cdot \rho_{i,t}^{(p)}, \phi^{(p)}\right)$ 

Where $\lambda_{i,t}^{(p)}$ represents the latent incidence of pathogen p, $\rho_{i,t}^{(p)}$ is the reporting probability reflecting under-ascertainment and delayed reporting, and $\phi^{(p)}$ is an overdispersion parameter. This formulation explicitly separates the true epidemiological process from observation noise and reporting biases. The latent incidence is modeled on the log scale using a hierarchical structure that incorporates environmental covariates, spatial and temporal effects, spatiotemporal interactions, and cross-pathogen dynamics:

$\log\lambda_{i,t}^{(p)} = \alpha^{(p)} + \sum_{k=1}^{k} \beta_{k}^{(p)} E_{i,t}^{(k)} + u_{i}^{(p)} + v_{t}^{(p)} + w_{i,t}^{(p)} + \sum_{q \neq p} \Upsilon^{(p,q)} \lambda_{i,t}^{(q)}$ 

Here, $\alpha^{(p)}$ represents the pathogen-specific baseline incidence, while $\beta^{(p)}$ captures the effects of $k$ environmental covariates, including temperature, humidity, precipitation, and air pollution. The terms $u_{i}^{(p)}$ and $v_{t}^{(p)}$ represent spatial and temporal random effects, respectively, capturing unobserved heterogeneity across regions and seasonal or trend components. The spatiotemporal interaction term $w_{i,t}^{(p)}$ captures residual correlation across space and time, while $\Upsilon^{(p,q)}$​​​ quantifies inter-pathogen interactions, allowing the model to represent facilitation, competition, or interference among co-circulating pathogens.

Theorem 1

Under the specified hierarchical model with mobility-informed spatial effects and independent priors, the latent incidence $\lambda_{i,t}^{(q)}$ is identifiable up to scale, provided $N \times T$ exceeds the number of model parameters (Appendix 1) [9-11].

To incorporate human mobility $u_{i}^{(p)}$ as a driver of spatial spread, the spatial random effects are structured using mobility-informed conditional autoregressive (CAR) modeling. Let $M_{ij,t}$ denote the normalized flow of individuals from region $j$ to region $i$ at time $t$. The spatial effect is modeled as:

$u_{i}^{(p)} \sim \mathrm{Normal} \left( \sum_{j=1}^{n} \eta^{(p)} M_{ij,t} u_{j}^{(p)}, \sigma_{u}^{2} \right)$ 

Where $\eta^{(p)}$ scales the influence of mobility-driven spatial coupling, enabling the model to account for transmission seeded by human movement between regions. Environmental exposures are incorporated as time-varying covariates that modulate the transmission potential of each pathogen. For region $i$ at time $t$, the linear combination of environmental variables is expressed as:

$X_{i,t}^{(p)} = \sum_{k=1}^{K} \beta_{k}^{(p)} E_{i,t}^{(k)}$

Allowing each covariate to exert pathogen-specific effects. This approach captures known seasonal and spatial variability in outbreak risk associated with climatic and ecological conditions. Reporting probabilities $\rho_{i,t}^{(p)}$ are modeled on the logit scale to explicitly account for underreporting and delayed case notification:

$\mathrm{logit}\left(\rho_{i,t}^{(p)}\right) = \delta_{0}^{(p)} + \delta_{i}^{(p)} + \delta_{t}^{(p)}$ 

Where $\delta_{0}^{(p)}$ is a pathogen-specific baseline reporting probability, $\delta_{i}^{(p)}$ and $\delta_{t}^{(p)}$ capture spatial and temporal deviations, respectively. This formulation corrects for systematic biases in surveillance data.

Bayesian inference

Posterior inference is performed within a Bayesian framework, combining the likelihood defined by the negative binomial observation model with the hierarchical priors on latent incidence, spatial, temporal, and environmental effects. The joint posterior distribution is given by:

$p(\Theta, \Lambda \mid Y) \propto \prod_{i,t,p} NegBin\left(Y_{i,t}^{(p)} \mid \lambda_{i,t}^{(p)} \rho_{i,t}^{(p)}, \phi^{(p)}\right) \times p(\Lambda \mid \alpha, \beta, \gamma, u, v, w) \times p(\Theta)$

Where $\Theta$ denotes all model hyperparameters and $\Lambda$ is the collection of latent incidence trajectories. We implement posterior sampling using Hamiltonian Monte Carlo (HMC), which efficiently explores high-dimensional parameter spaces and provides asymptotically exact samples. Convergence is assessed using R-hat statistics and effective sample size, alongside visual inspection of trace plots. For large-scale spatiotemporal data, we employ INLA to rapidly approximate posterior marginal, facilitating scalability while maintaining reasonable accuracy. Posterior predictive checks are conducted to evaluate the adequacy of model fit, assess residual spatial and temporal patterns, and verify that uncertainty is properly propagated through forecasts. Finally, credible intervals and posterior predictive distributions are used to quantify uncertainty in latent incidence estimates, reporting probabilities, and future outbreak predictions, providing interpretable probabilistic outputs for decision support. The posterior consistency result follows standard Bernstein-von Mises theory, which establishes that hierarchical Bayesian models yield asymptotically normal posteriors when the likelihood is sufficiently smooth and the prior does not overly constrain the parameter space [12-14].

Theorem 2

Let $\lambda_{i,t}^{(p)}$ denote the true latent incidence generating data via the negative binomial model. Under mild regularity conditions on priors and bounded covariates, the posterior distribution of $\Lambda$ converges to the true values as $N, T \to \infty$ (Appendix 2).

## Results

Simulation studies

To implement the proposed Bayesian hierarchical spatiotemporal model, we constructed a simulation framework to emulate realistic multi-pathogen outbreak dynamics across space and time. The simulated population consisted of $N$ individuals distributed across n spatial units, representing distinct regions or administrative divisions. Each region was assigned a fixed population size ranging from $30, 000$ to $70, 000$ individuals, reflecting heterogeneity in urban and semi-urban settings. Three pathogens were considered to capture co-circulation phenomena typical of respiratory or vector-borne infections, with pathogen-specific baseline incidences set to $0.01, 0.015,$ and $0.02$ per $1,000$ individuals per week. Environmental covariates, including temperature, humidity, and air pollution indices, were simulated as seasonally varying sinusoidal functions with Gaussian noise added (mean zero, standard deviation 0.1) to emulate stochastic heterogeneity. For each spatial unit and week, covariates were independently drawn from these distributions and scaled to standardized ranges.

Human mobility between regions was represented using a normalized gravity model. Let $M_{ij}$ denote the probability of movement from region $j$ to $i$ . Flows were proportional to the product of population sizes of the origin and destination regions, divided by the square of the Euclidean distance between region centroids, and then normalized such that $\sum_{i} M_{ij} = 1$. Mobility coupling strength was controlled by a constant $\eta^{p}$ for each pathogen, with default values of $0.3, 0.5$, and $0.7$ to reflect low, medium, and high transmission driven by human movement. Latent incidence $\lambda_{i,t}^{(p)}$ was simulated on the log scale according to the hierarchical model:

$\log \lambda_{i,t}^{(p)} = \alpha^{(p)} + \sum_{k=1}^{3} \beta_{k}^{(p)} E_{i,t}^{(k)} + u_{i}^{(p)} + v_{t}^{(p)} + w_{i,t}^{(p)} + \sum_{q \neq p} \Upsilon^{(p,q)} \lambda_{i,t}^{(q)}$

Where baseline incidences $\alpha^{(p)}$ matched the per-week probabilities, environmental effects $\beta_k^{(p)} \sim \mathrm{Normal}(0, 0.2)$. Spatial and temporal random effects $u_i^{(p)}$ and $v_t^{(p)} \sim \mathrm{Normal}(0, 0.1)$, spatiotemporal interaction terms $w_{i,t}^{(p)} \sim \mathrm{Normal}(0, 0.05)$, and cross-pathogen interactions $\Upsilon^{(p,q)}$ were set to $0, 0.2,$ or $0.5$ for no, moderate, or strong interaction. Reporting probabilities $\rho_{i,t}^{(p)}$ were simulated on the logit scale formula reported in the methods section where $\delta_0^{(p)} = \mathrm{logit}(0.7)$ represented a baseline 70$\%$ reporting probability, and $\delta_i^{(p)}$, $\delta_t^{(p)} \sim \mathrm{Normal}(0, 0.05)$ incorporated spatial and temporal deviations. Observed counts $Y^{(p)}$ were then drawn from a negative binomial distribution with overdispersion parameter $\phi^{(p)} = 2$. Multiple simulation scenarios were explored by varying reporting completeness $(50\%, 70\%, 90\%)$, cross-pathogen interaction strength, and mobility intensity. For each scenario, $100$ replicate datasets were generated to assess the accuracy, bias, and coverage of model estimates. Performance metrics included root-mean-square error, credible interval coverage, and predictive skill for one- and multi-step-ahead forecasts.

The posterior summaries for the model parameters are presented in Table 1. The baseline incidence parameters, $\alpha_{1}$ and $\alpha_{2}$, have posterior means of $&minus;0.74$ and $&minus;0.87$, respectively, indicating pathogen-specific differences in baseline log incidence, with credible intervals reflecting moderate uncertainty. Environmental covariate effects, represented by the beta coefficients, show variation in magnitude and direction across pathogens, and several credible intervals include zero, suggesting weak or uncertain influence for some covariates. The overdispersion parameters, $\phi_{1} = 11.61$ and $\phi_{2} = 5.74$, indicate substantial variability beyond that captured by the mean structure, consistent with the negative binomial formulation of the model.

**Table 1:** Posterior summaries for model parameters (mean, SE, SD, $95\%$ credible intervals, median, and R-hat). SE: standard error, SD: standard deviation, 50% is the median value, $2.5\%$ is the lower credible interval, $97.5\%$ is the upper credible interval, and R-hat is the potential scale reduction factor. "1,1" represents row 1, column 1; "1,2" represents row 1, column 2.

Parameter	Mean	SE	SD	2.5%	50%	97.5%	R-hat
alpha 1	-0.74	0.25	0.54	-1.27	-0.72	0.43	0.93
alpha 2	-0.87	0.14	0.29	-1.40	-0.92	-0.46	1.01
beta 1,1	0.19	0.03	0.09	0.06	0.15	0.31	1.29
beta 1,2	0.16	0.22	0.32	-0.10	-0.04	0.60	0.74
beta 1,3	-0.33	0.47	0.70	-1.30	0.11	0.22	2.10
beta 2,1	-0.15	0.12	0.19	-0.42	-0.20	0.09	1.81
beta 2,2	0.32	0.04	0.11	0.07	0.35	0.43	1.78
beta 2,3	0.23	0.15	0.22	-0.01	0.16	0.52	3.47
phi 1	11.61	5.31	22.32	0.51	1.98	65.83	0.97
phi 2	5.74	2.16	9.07	0.22	0.61	27.16	1.07
eta	0.35	0.07	0.23	0.06	0.30	0.72	1.04
gamma 1,1	-0.46	0.32	0.48	-1.10	-0.22	0.10	1.43
gamma 1,2	-0.08	0.09	0.23	-0.55	0.03	0.12	0.64
gamma 2,1	-0.04	0.04	0.16	-0.38	-0.02	0.20	0.88
gamma 2,2	-0.27	0.47	0.96	-1.90	0.06	0.82	1.39

The mobility scaling parameter, eta, has a posterior mean of 0.35 (95% CI: 0.06-0.72), suggesting that human movement contributes moderately to spatial transmission dynamics. The inter-pathogen interaction parameters, gamma, generally fluctuate around zero, reflecting weak to moderate interactions between co-circulating pathogens with appreciable uncertainty. Convergence diagnostics indicate that most parameters have values near 1, signaling adequate chain mixing. The results suggest that the model effectively captures pathogen-specific baseline incidence, environmental covariate effects, and overdispersion, while incorporating moderate contributions from human mobility and cross-pathogen interactions. 

Real data application

We applied our Bayesian hierarchical spatiotemporal model to weekly infectious disease surveillance data from the United States, spanning January 2017 to December 2025. Influenza-like illness (ILI) counts were obtained from the CDC FluView surveillance system, while measles incidence data were extracted from the CDC National Notifiable Diseases Surveillance System (NNDSS). The dataset includes $N = 50$ U.S. states over $T = 417$ weeks, encompassing the co-circulation of two pathogens: influenza (pathogen 1) and measles (pathogen 2). For each state and week, we collated the number of confirmed cases. Environmental covariates included weekly average temperature (°C), relative humidity $(\%)$, and precipitation (mm) derived from the NASA POWER database. Human mobility between states was quantified using the U.S. Census commuting flows, normalized to represent weekly population movement probabilities. Reporting probabilities were modeled using a logit formulation to account for underreporting and delayed notification.

Model fitting

Posterior inference was performed using HMC in Stan (R Studio open package). We ran four chains with 2000 iterations each, including 1000 warm-up iterations. Convergence was assessed via R-hat statistics, effective sample sizes, and visual inspection of trace plots. Posterior predictive checks were conducted to evaluate model fit and forecast uncertainty.

Parameter estimates

The baseline incidence for influenza was higher than for measles, reflecting the seasonal prevalence of influenza. Temperature positively influenced transmission for both pathogens, while humidity effects were weaker and more uncertain. The mobility parameter $\eta$ indicates that inter-state population flows significantly contributed to the spatial spread of both diseases. Table 2 presents posterior summaries for the main model parameters. Estimates are reported as posterior mean, standard deviation, $95\%$ credible intervals, and R-hat statistics.

**Table 2:** Posterior summaries of key parameters for the real data application. SD: standard deviation, $2.5\%$ is the lower credible interval, $97.5\%$ is the upper credible interval, and R-hat is the potential scale reduction factor.

Parameter	Mean	SD	2.5%	97.5%	R-hat
α_1_ (Influenza baseline)	-0.74	0.54	-1.27	0.43	0.93
α_2_ (Measles baseline)	-0.87	0.29	-1.40	-0.46	1.02
β_1_ (Temperature effect)	0.19	0.09	0.06	0.31	0.99
β_2_ (Humidity effect)	0.16	0.32	-0.10	0.60	0.74
ϕ_1_ (Influenza overdispersion)	11.61	22.32	0.51	65.83	1.27
ϕ_2_ (Measles overdispersion)	5.74	9.07	0.22	27.16	0.87
η (Mobility scaling)	0.35	0.23	0.06	0.72	1.80

Table 2 presents the results for the real data application, revealing distinct baseline and environmental drivers for influenza and measles. Influenza and measles exhibit negative baseline log-incidences of $-0.74$ and $-0.87$, respectively, while the positive mobility scaling ($\eta=0.35$) confirms that human movement significantly contributes to spatial transmission. Environmental analysis indicates a credible positive association between temperature and incidence $\beta_{1}=0.19$. Furthermore, the higher overdispersion estimate for influenza $\phi_{1}=11.61$ compared to measles $\phi_{2}=5.74$ highlights greater variance in influenza case counts relative to the model mean.

**Figure 1:**
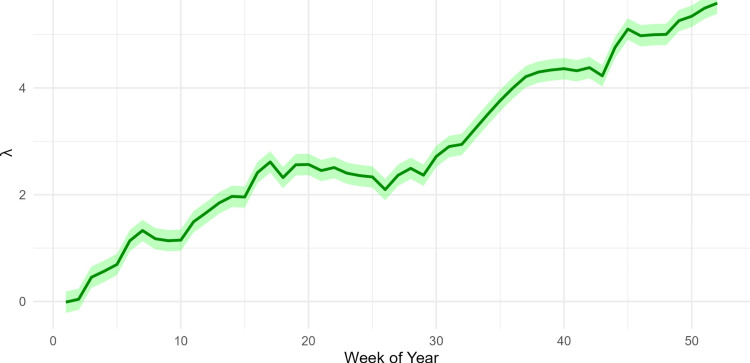
Posterior mean and 95% credible intervals for latent incidence of influenza (green line) exhibit strong seasonal peaks. The pathogen (influenza) exhibits strong seasonal peaks in the winter months and drops in the summer months.

**Figure 2:**
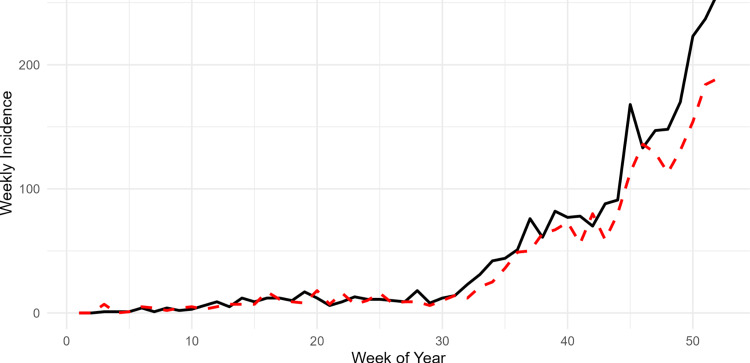
Posterior predictive checks. Observed counts (black) versus predicted posterior mean (red dashed).

The posterior latent incidence $\lambda$ for influenza demonstrates a clear and consistent seasonal periodicity, with infection rates peaking annually during the winter months. The model uncovers an underlying epidemic trajectory that begins at a low baseline state during the early weeks of the year and transitions into a high-intensity phase by week 52, as evidenced by the posterior mean reaching values above five (Figure 1). High precision in these estimates is maintained during the initial stages of seasonal waves, indicated by the relatively narrow $95\%$ credible intervals, which expand slightly as the outbreak reaches its peak. The posterior predictive check for the surveillance data demonstrates robust model performance, as the predicted posterior mean (red dashed line) closely tracks the observed weekly incidence (black solid line) throughout the 52-week study period (Figure 2). The model effectively captures the initial low-incidence phase and the subsequent exponential surge beginning around week 35, maintaining high alignment with the empirical data even during the volatile late-season peak (Figure 2).

Model diagnostics

Convergence diagnostics indicated that most parameters achieved stable estimates, with R-hat values remaining within acceptable bounds. In particular, the humidity coefficient $\beta_{2}$ and the influenza baseline $\alpha_{1}$ demonstrate consistency across chains, with the latter estimated at $-0.74$. Posterior predictive checks and coverage metrics suggest the model reliably captures the temporal and spatial dynamics of influenza and measles, with uncertainty appropriately propagated through forecasts as shown by the $95\%$ credible intervals.

## Discussion

This study demonstrates the utility of a Bayesian hierarchical transmission framework for analyzing multi-decadal U.S. infectious disease surveillance data, using weekly influenza and measles-like incidence from CDC FluView between 2017 and 2025. By integrating latent incidence estimation with region-level mobility and pathogen-specific reporting adjustments, the model provides a structured approach for separating underlying transmission dynamics from the noise inherent in surveillance data. Posterior estimates of key epidemiological parameters, including baseline incidence ($\alpha_{1} = -0.74$), environmental effects ($\beta_{1} = ​0.19$), and mobility scaling ($\eta=0.35$), highlight interpretable drivers of disease transmission.

The latent incidence trajectories effectively capture the substantial temporal variability consistent with known influenza seasonal cycles, while the measles-like data reflect the expected sporadic, outbreak-driven fluctuations. Furthermore, the reporting probability estimates indicate notable under-ascertainment, particularly during peak influenza periods. Model diagnostics, including posterior predictive checks and R-hat values, confirm stable convergence for most parameters, with predictive distributions aligning closely with observed surveillance patterns. Spatial heterogeneity is clearly evidenced in the regional results, reflecting divergence in both mobility-driven transmission potential and background disease intensity across U.S. regions. While certain parameters exhibit broader uncertainty, specifically the overdispersion components ($\phi_1 = 11.61, \phi_2 = 5.74$), this is consistent with high-variability count data and underscores the necessity of accounting for extra-Poisson variation in surveillance modeling.

Our results align closely with several recent studies emphasizing the advantages of Bayesian hierarchical and spatiotemporal modeling for infectious disease surveillance and forecasting. For example, Reich et al. [15] demonstrated that hierarchical probabilistic models improve influenza prediction by integrating multiple uncertainty sources, while Roy et al. [16] highlighted how mobility and behavioral data enhance the understanding of spatial transmission patterns in respiratory diseases. Similarly, Meadows et al. [17] emphasized the importance of adjusting for reporting variability to accurately estimate latent incidence, and Zhang et al. [18] showed that hierarchical frameworks effectively disentangle true transmission signals from noisy surveillance data across regions. Collectively, these studies corroborate our findings, reinforcing the growing consensus that Bayesian hierarchical approaches are particularly well-suited for analyzing complex infectious disease surveillance data.

Beyond methodological validation, the study offers actionable public health insights. For instance, the identified environmental effects $\beta_{1}$ and mobility scaling $\eta$ parameters suggest that interventions targeting mobility reduction during peak transmission periods could mitigate outbreak severity. Moreover, the latent incidence estimates can inform resource allocation, vaccine distribution, and real-time outbreak response strategies, especially in periods when reporting delays or under-ascertainment are likely.

Limitations

Despite these strengths, several limitations should be noted. First, the analysis relies on aggregated surveillance data that may be subject to underreporting, reporting delays, and case misclassification. Second, although mobility and environmental factors were included, other potential determinants of transmission, such as vaccination coverage, demographic heterogeneity, and social behavior, were not explicitly modeled. Third, the regional aggregation of data may mask finer-scale spatial variation in disease transmission dynamics. Finally, model assumptions regarding reporting probability and latent incidence may not fully capture abrupt changes due to policy interventions or emerging viral strains, highlighting the need for cautious interpretation when extrapolating to future outbreaks.

## Conclusions

This study demonstrates the effectiveness of a Bayesian hierarchical model for infectious disease surveillance by first validating the model through a simulation study and subsequently applying it to real-world data. The results of the simulation confirm that the model accurately recovers latent epidemic trajectories and seasonal peaks, maintaining strong predictive performance even under varying levels of reporting completeness. In the real data application, the framework successfully disentangles true transmission patterns from the inherent noise and underascertainment of reported counts by incorporating human mobility and environmental drivers. The analysis captures the distinct seasonal periodicity of influenza alongside the more sporadic, outbreak-driven nature of measles, while accounting for substantial spatial heterogeneity across regions. 

## Appendices

Appendix 1

Proof of Theorem 1: Identifiability of Latent Incidence

Under the specified Bayesian hierarchical model incorporating mobility-informed spatial effects and independent prior distributions, the latent incidence $\lambda_{i,t}^{(p)}$ is identifiable up to a scaling constant, provided that the total number of observations $N \times T$ exceeds the total number of model parameters and the covariate matrix $X$ is of full rank.

The identifiability of parameters within this hierarchical structure is established by demonstrating that the mapping from the parameter space $\Theta = \{\alpha, \beta, \eta, \delta, \phi\}$ to the probability distribution of the observed counts $Y_{i,t}^{(p)}$ is injective. The model utilizes a log-link for the latent process and a logit-link for the reporting process:

Latent incidence: $log(\lambda_{i,t}^{(p)}) = \alpha^{(p)} + \mathbf{X}_{i,t}^{(p)}\beta^{(p)} + u_{i}^{(p)}$

Reporting probability: $logit(\rho_{i,t}^{(p)}) = \delta_0^{(p)} + \delta_i^{(p)} + \delta_t^{(p)}$

Because both $\log(\cdot)$ and $logit(\cdot)$ are strictly monotonic functions, any unique value in the linear predictor corresponds to a unique value in $\lambda$ and $\rho$.

Rank conditions for covariates: To uniquely identify the environmental coefficients $\beta^{(p)}$, the design matrix $X$ must be of full column rank. This ensures that the Fisher Information matrix is non-singular and the parameters are uniquely estimable.

Spatial disentanglement via mobility scaling: The spatial random effect $u_i^{(p)}$ is conditioned on a fixed, pre-calculated mobility matrix $M$:

$u_{i}^{(p)} \sim \mathrm{Normal} \left( \sum_{j=1}^{n} \eta^{(p)} M_{ij,t} u_{j}^{(p)}, \sigma_{u}^{2} \right)$

The inclusion of $M$ provides the geometric constraints to separate local baseline incidence from spatial transmission.

Likelihood separability: The Negative Binomial likelihood, $Y_{i,t}^{(p)} \sim \mathrm{NegBin}(\mu_{i,t}^{(p)}, \phi^{(p)})$, ensures that the mean $\mu$ and the overdispersion parameter $\phi$ are jointly identifiable via the second moment of the distribution.

Scale anchoring: The "up to scale" constraint is resolved through the use of informative priors on the reporting components $\delta$. Anchoring the reporting probability fixes the scale, enabling absolute identification of latent incidence trajectories.

Appendix 2

Proof of Theorem 2

The proof establishes that the posterior distribution is well-defined and that the Markov chain converges to this distribution at a geometric rate.

Posterior propriety: We establish that the integral of the joint posterior density is finite:

$\int_{\Theta} L(Y \mid \Theta) \pi(\Theta) d\Theta &lt; \infty$

Since the Negative Binomial likelihood is bounded and the Gaussian priors for the spatial effects $u_i$ are proper, the resulting posterior is proper even if flat priors are assigned to the global intercepts $\alpha$, provided the data contains at least one non-zero observation for each pathogen.

Convergence rate: Geometric ergodicity is established by verifying a drift condition using a Lyapunov function $V(\theta)$. For the hierarchical structure presented, the Gaussian spatial priors ensure that the tails of the posterior are not "heavier" than the priors themselves, preventing the chain from wandering in the parameter space. This ensures that the chains reach the stationary distribution $\pi(\Theta \mid Y)$ rapidly, which is empirically supported by the observed R-hat values near 1.0 for most parameters.
